# Correlation among isolated teratozoospermia, sperm DNA fragmentation and markers of systemic inflammation in primary infertile men

**DOI:** 10.1371/journal.pone.0251608

**Published:** 2021-06-07

**Authors:** Luigi Candela, Luca Boeri, Paolo Capogrosso, Walter Cazzaniga, Edoardo Pozzi, Federico Belladelli, Andrea Baudo, Andrea Ravizzoli, Eugenio Ventimiglia, Paola Viganò, Massimo Alfano, Costantino Abbate, Julian Cornelius, Agostino Mattei, Francesco Montorsi, Andrea Salonia

**Affiliations:** 1 Division of Experimental Oncology/Unit of Urology, URI, IRCCS Ospedale San Raffaele, Milan, Italy; 2 University Vita-Salute San Raffaele, Milan, Italy; 3 Department of Urology, Foundation IRCCS Ca’ Granda–Ospedale Maggiore Policlinico, University of Milan, Milan, Italy; 4 Department of Urology and Andrology, Ospedale di Circolo and Macchi Foundation,Varese, Italy; 5 Obstetrics and Gynaecology Department, IRCCS Ospedale San Raffaele, Milan, Italy; 6 Division of Genetics and Cell Biology, Reproductive Sciences Laboratory, IRCCS Ospedale San Raffaele, Milan, Italy; 7 Department of Urology, Luzerner Kantonsspital, Lucerne, Switzerland; University Hospital of Münster, GERMANY

## Abstract

**Aim:**

To assess the prevalence of isolated teratozoospermia (iTZS) in a cohort of infertile and fertile men; explore the relationship between iTZS, inflammatory parameters and sperm DNA fragmentation index (SDF) in the same cohort.

**Materials and methods:**

1824 infertile men and 103 fertile controls. Semen analysis, the neutrophil-to-lymphocyte ratio (NLR) and serum hormones were investigated. DFI was tested in infertile men only. According to 2010 WHO semen analysis, patients were categorized in 3 sub-groups of isolated sperm defects: isolated oligozoospermia (iOZS), isolated asthenozoospermia (iAZS) and iTZS. Descriptive statistics and linear regression models tested the association between clinical variables and inflammatory markers.

**Results:**

Among infertile men, iAZS, iTZS, and iOZS were found in 13.9%, 11.9% and 4.1% participants, respectively. iTZS was found in 37 (35.9%) fertile men. Infertile men with iTZS had higher NLR values than those with iOZS, iAZS and men with normal semen parameters (all p<0.001). FSH and LH were higher and inhibin B lower in iOZS infertile men compared to all other groups (p≤0.001). Hormonal characteristics were similar between iTZS infertile and fertile men. Similarly, iTZS infertile men had higher SDF than all other groups (all p<0.001). Infertile men with iTZS had higher NLR values than fertile men with iTZS (p<0.01). Linear regression analysis showed that, in infertile men, iTZS was associated with SDF and NLR (all p≤0.01).

**Conclusions:**

iTZS was found in 11.9% of infertile men but it was even more prevalent in fertile controls. Infertile men with iTZS had higher NLR than fertile controls and increased SDF values than infertile participant with iAZS, iOZS, or normal semen parameters. No differences in hormonal characteristics were found between infertile and fertile men with iTZS.

## Introduction

Couple’s infertility is a rising issue worldwide with a male factor infertility (MFI) contributing to approximately half of the cases [1]. Furthermore, epidemiological studies have shown a tremendous decrease in sperm quality over the last decades in the general male population (50–60% decline of sperm count) [2–4]. In this context, men with MFI should always undergo a detailed evaluation, thus including medical and reproductive history, an accurate physical examination and a semen analysis [1, 5].

Teratozoospermia is the result of a defective cell differentiation during spermatogenesis and it has been associated with several genetic and environmental factors as well as advanced paternal age and psychological distress [6–9]. Teratozoospermia occurs frequently in combination with oligozoospermia (OZS) (i.e., <15 million spermatozoa/mL and < 39 million sperm / ejaculate) and asthenozoospermia (AZS) (i.e., <32% progressive motile spermatozoa), thus defining the oligo-astheno-teratozoospermia (OAT) syndrome [5]. However, even isolated TZS (iTZS), depicted as the only impaired parameter at semen analysis, is a relatively common finding in infertile men [10, 11].

As a whole, iTZS has been extensively associated with both spontaneous pregnancy and fertilization outcomes at assisted reproductive technology (ART), but results are mostly inconclusive [9, 10, 12]. Moreover, previous studies have investigated the relationship between iTZS and sperm DNA damage in males from the general population and in those with MFI [13–15]. Infertile men with iTZS showed greater prevalence of chromosomal abnormalities and higher DNA fragmentation indexes compared to controls [15, 16].

Abnormal sperm morphology was also linked to increased levels of reactive oxygen species (ROS) in semen, thus contributing to sperm DNA damage [13, 14]. Overall, published literature suggests that infertile men with iTZS have higher rates of sperm DNA fragmentation and imbalanced oxidative stress (OS) status than fertile controls. Conversely, rates of DNA alterations of iTZS patients compared to men with different isolated sperm abnormalities have never been comprehensively investigated. Likewise, inflammation parameters which may recapitulate the inflammatory status—such as the neutrophil-to-lymphocyte ratio (NLR), the monocyte-to-eosinophil ratio (MER), and the platelet-to-lymphocyte ratio (PLR)—have been recently associated with various andrological diseases [17, 18], but never with iTZS in infertile men.

Thereof, with the specific hypothesis that iTZS condition can be considered as a useful and effective marker of the state of OS, inflammation and, consequently, of sperm DNA damage, in daily clinical practice and throughout the diagnostic work-up of infertile patients, we aimed i) to evaluate the prevalence of iTZS in a homogeneous cohort of white-European men searching medical help for couple’s infertility compared to a cohort of same-race fertile controls; ii) to compare inflammatory markers of iTZS infertile men with those of the cohort of fertile controls; and, iii) to investigate a potential association between inflammatory markers and sperm DNA damage and iTZS compared to other isolated sperm abnormalities.

## Materials and methods

Data from 2035 white-European men evaluated at a single academic centre for couple’s infertility between September 2010 and September 2019 were retrospectively analysed. Infertility was defined as not conceiving a pregnancy after one year or more of unprotected sexual intercourses regardless of whether or not a pregnancy ultimately occurs, as for WHO criteria [19]. Patients were considered if they were 18–55 years old and had either pure MFI or mixed factor infertility. MFI was defined after a global diagnostic evaluation of the female partners.

Similarly, we collected data from 103 fertile controls of comparable age (i.e., men who had fathered at least one child, spontaneously conceived, with a time-to-pregnancy within 12 months, as for WHO criteria). Fertile men were recruited via their partners who had been expectant and new mothers at our department of Obstetrics and Gynaecology during the hospital stay and underwent the same investigation of infertile participants, except for the genetic tests, according to our internal research protocol.

### Clinical features

Participants were assessed with an accurate and complete medical history. Health comorbidities were scored with the Charlson Comorbidity Index (CCI) [20]. Equally, weight and height were measured, calculating body mass index (BMI; kg/m^2^) for each participant. Testes volume was measured in all cases using Prader’s orchidometer estimation by the same expert andrologist and we calculated the mean value among the two sides. Varicocele was clinically evaluated in every man. Smoking addiction was investigated in each case and then categorized into: no smokers/former smokers and active smokers.

### Serum parameters

Venous blood samples were drawn during the morning (between 7 AM and 11 AM) after an overnight fast. In all participants, follicle-stimulating hormone (FSH), luteinizing hormone (LH) and total testosterone (tT) were measured by a direct chemiluminescence immunoassay (CLIA). Sex hormone-binding globulin (SHBG) was measured by an electrochemiluminescence assay (ECLIA). Conversely, inhibin B (InhB) was measured by Enzyme-Linked ImmunoSorbent Assay (ELISA).

Moreover, blood testing included total and differential white blood cell (WBC) counts. Total counts for WBC, neutrophils, lymphocytes and monocytes were assessed using an automated blood cell counter. Moreover, NLR, MER and PLR were calculated as ratios from complete blood count in every participant. As per our local guidelines, karyotype analysis, along with tests for Y-chromosome microdeletions and cystic fibrosis mutations were obtained for every infertile man [21].

### Semen analysis and sperm DNA fragmentation

At least two consecutive semen analyses were requested for every participant [5]. According to WHO criteria, semen samples were collected after a sexual abstinence of 2–5 days and analyzed within 2 h of ejaculation. Semen volume, sperm concentration, total sperm count in the ejaculate, progressive sperm motility and normal sperm morphology were considered for the current study.

The improved Neubauer hemocytometer chamber (100-μm-deep; Brand™ Blaubrand™ Neubauer Improved Counting Chambers, Fisher Scientific, Loughborough, UK) was used for the calculation of sperm concentration and total sperm count in the ejaculate. Sperm morphology was assessed preparing a smear of semen on stain-coated Test-simplets slides (Waldeck GmbH & Co. KG) and examinating the slide with brightfield optics at ×1000 magnification (Nikon Eclipse E 200, Nikon Instruments Europe B.V., Rome, Italy) with oil immersion within 1 hour of slide preparation. The evaluation of the percentage of morphologically normal and abnormal spermatozoa was performed according to the WHO guidelines and approximately 200 spermatozoa per replicate were assessed. Sperm motility was assessed by mixing twice the sample, using a wet preparation of 20 microM deep for each replicate, by examining the slide with phase-contrast optics at ×200 magnification and by assessing approximately 200 spermatozoa per replicate for the percentage of different motile categories. Patients with azoospermia (any type) were not considered as per exclusion criteria.

Sperm DNA fragmentation (SDF) index, as measured by sperm chromatin structure assay (SCSA), was assessed only in infertile men, considering a pathologic SDF threshold of ≥30% [1, 22, 23]. The same laboratory was used for the analyses of all parameters.

### Study populations

For the objectives of the current study, we identified 3 subgroups of patients with isolated sperm abnormalities according to WHO reference values: isolated oligozoospermia (iOZS: <15 million sperm per ml and < 39 million sperm / ejaculate, ≥32% progressive motility and ≥4% normal forms); isolated AZS (iAZS: <32% progressive motility, ≥15 million sperm/ml and ≥4% normal forms); and, iTZS (<4% normal forms, ≥15 million sperm/ml and ≥32% progressive motility).

We excluded 211 (10.4%) primary infertile individuals because they missed one or more of the entry criteria [history of cryptorchidism (*n* = 25; 1.2%); abnormal genetic tests (any type) (*n* = 29; 1.4%); signs or symptoms suggestive for infections of the genitourinary system (*n* = 15; 0.7%) or positive semen or urine cultures (*n* = 137; 6.7%); a history of infertility treatment in the preceding year (*n* = 5; 0.2%)]. A cohort of 1824 infertile men and 103 fertile controls was considered for the first part of statistical analyses.

Of note, SDF and complete blood count were included in the routine baseline investigation of infertile men only over the last 5 years. Hence, we included in the second part of the statistical analyses only those participants with valid values for SDF, NLR, PLR and MER. Therefore, a cohort of 365 infertile men was compared to the 103 fertile controls with the aim to investigate the association between isolated semen parameters and inflammatory biomarkers in the whole cohort and SDF in infertile men only.

Data collection respected the principles described in the Declaration of Helsinki. All participants signed an informed consent agreeing to share their own anonymous information for future studies. The current study was authorized by the IRCCS San Raffaele Hospital Ethical Committee (Prot. 2014-Pazienti Ambulatoriali).

### Statistical methods

Normality of data was tested with the Shapiro–Wilk test. Data are presented as medians (interquartile range; IQR) or frequencies (proportions). First main outcome of the study was to investigate the rate of men with normal semen parameters, isolated sperm abnormalities, or ≥2 semen abnormalities among the whole cohort of participants. Second outcome was to conduct a sub-analysis only considering those participants (*n* = 468; 24.3%) valid values for SDF, NLR, PLR and MER. In this sub-cohort, the Kruskal Wallis test and the Fisher exact test were applied to compare clinical and demographics characteristics, hormonal and semen parameters between men with normal semen parameters and those with isolated semen abnormalities. Spearman’s correlation analysis tested the association between SDF and inflammatory biomarkers with clinical variables. Similarly, we applied descriptive statistics to compare clinical characteristics, laboratory values and semen parameters between infertile and fertile men with iTZS. Univariable (UVA) and multivariable (MVA) linear regression models were used to identify variables (e.g., age, BMI, CCI, FSH, smoking status, sperm count and isolated semen abnormalities) associated with SDF and NLR in infertile men.

Statistical analyses were performed using SPSS v.26 (IBM Corp., Armonk, NY, USA). All tests were two sided, and statistical significance level was determined at p < 0.05.

## Results

Of 1824, iAZS, iTZS, and iOZS was found in 253 (13.9%), 217 (11.9%) and 75 (4.1%) infertile patients, respectively (Table 1). Two or more semen abnormalities were found in 998 (54.7%) infertile men, whereas 281 (15.4%) patients had normal semen parameters. Of 103 fertile controls, 37 (35.9%) men had iTZS, 1 (1.0%) had iAZS, and 23 (22.3%) men presented with two or more semen abnormalities; 42 (40.8%) fertile controls had normal semen parameters. In fertile men, median (IQR) sperm concentration, progressive motility and normal morphology were 50 (26.7–70) x10^6^/mL, 46 (34–53) % and 2 (1–10) %, respectively. Isolated sperm abnormalities were more frequently found in fertile than infertile (36.9% vs. 29.9%) men; conversely, either 2 alterations or OAT were more prevalent in infertile men (p≤0.001) (Table 1).

**Table 1:** Semen characteristics among the whole cohort according to fertility status [No. (%)] (No. = 1927).

	Fertile [103 (5.3%)]	Infertile [1824 (94.7%)]		Fertile	Infertile	p value
Normal semen parameters	42 (40.8)	281 (15.4)	Normal semen parameters	42 (40.8)	281 (15.4)	≤0.001
Isolated teratozoospermia	37 (35.9)	217 (11.9)	Isolated sperm abnormalities	38 (36.9)	545 (29.9)	
Isolated oligozoospermia	0 (0.0)	75 (4.1)	2 semen abnormalities	18 (17.5)	555 (30.4)	
Isolated asthenozoospermia	1 (1.0)	253 (13.9)	OAT	5 (4.9)	443 (24.3)	
2 semen abnormalities	18 (17.5)	555 (30.4)				
OAT	5 (4.9)	443 (24.2)				

Keys: OAT = oligo-astheno-teratozoospermia; * p value according to the Fisher exact test

**Fig 1:**
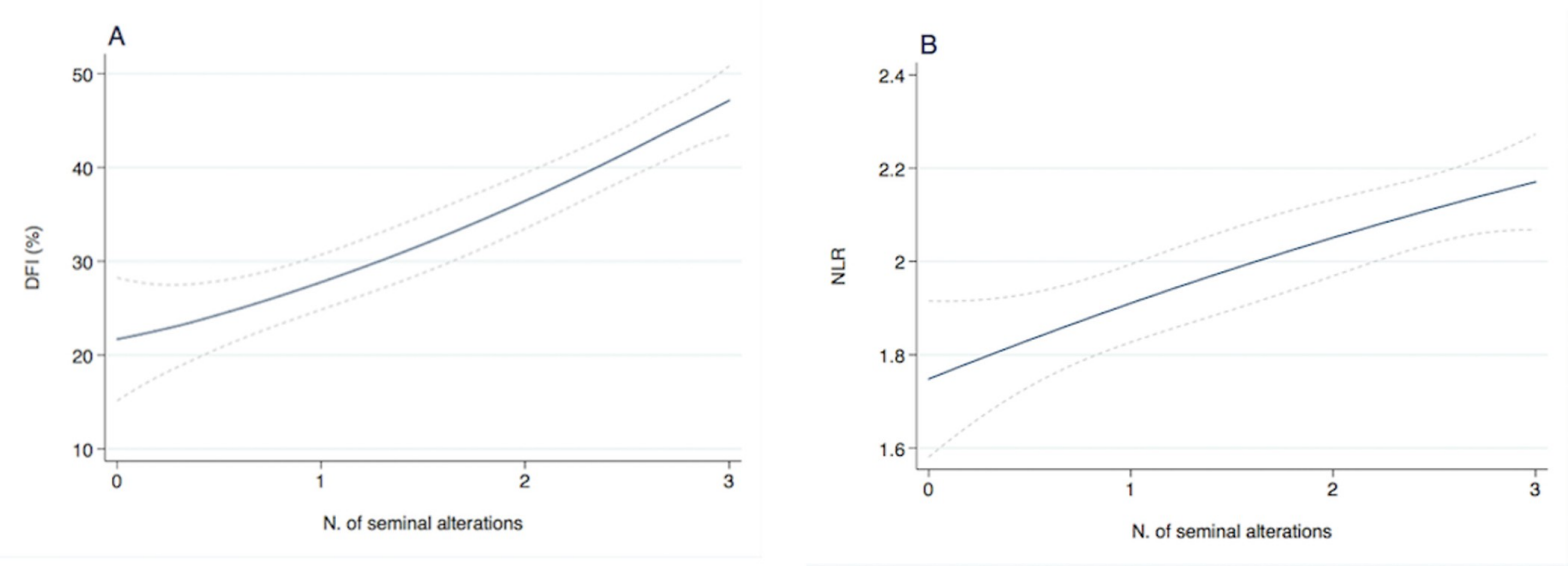
Estimated sperm DNA fragmentation (SDF) (A) and neutrophil-to-lymphocyte ratio (NLR) (B) according to the number of sperm alterations in infertile men. Dashed lines represent 95% CI.

The association between semen parameters, SDF and inflammatory biomarkers was analyzed in 468 participants. Fig 1A and 1B graphically depicts the linear increase of SDF and NLR with the number of sperm abnormalities in the cohort of infertile men. Conversely, no association was found between NLR and sperm abnormalities in fertile men.

Age, BMI, CCI, tT and SHBG were similar among infertile men as segregated according to semen parameter’s alterations (Table 2). Serum FSH and LH were higher in infertile men with iOZS compared to those in all other groups (all p≤0.01). Conversely, inhibin B was lower in iOZS compared to iAZS, iTZS and the group of men with normal semen parameters (all p = 0.01) (Table 2). NLR values were higher in patients with iTZS than in those with iOZS, iAZS and in men with normal semen parameters (all p<0.001). Similarly, higher median SDF (all p<0.001) and greater rates of SDF >30% (p = 0.02) were more frequently observed in iTZS than in all other groups (Table 2).

**Table 2:** Descriptive statistics of the cohort of infertile patient units (No. = 365).

	Normal semen parameters	iTZS	iOZS	iAZS	p-value
No. of individuals	104 (28.5%)	106 (29.0%)	35 (9.6%)	120 (32.9%)	
Age (years)					0.43
Median (IQR)	36.0 (33–40)	37.0 (34.0–41)	38.0 (34.0–42)	37.0 (35.0–41)	
Range	18–50	19–54	19–47	19–55	
BMI (kg/m^2^)					0.12
Median (IQR)	24.2 (22.8–26.4)	24.6(23–26.5)	25.2 (23.6–27.6)	25.2 (23.3–27.1)	
Range	18.6–41.6	19.5–37.7	20.6–39.1	20.3–34.7	
CCI (score)					0.42
Median (IQR)	0.0 (0.0)	0.0 (0.0)	0.0 (0.0)	0.0 (0.0)	
Median (SD)	0.1 (0.7)	0.1 (0.3)	0.1 (0.2)	0.1 (0.3)	
Range	0–3	0–2	0–3	0–4	
CCI ≥ 1 [No. (%)]	5 (4.8)	9 (8.5)	1 (2.9)	11 (9.2)	0.41
Total testis volume (Prader estimation)					0.01
Median (IQR)	20.0 (15–25)	20.0 (15–25) ^#^	15.0 (12–20) ^§^^,^^†^	20.0 (15–25)	
Range	8–25	10–25	8–25	8–25	
Smoking status [No. (%)]					0.33
No smokers/Former smokers	80 (76.9)	72 (67.9)	24 (68.6)	92 (76.7)	
Active smokers	24 (23.1)	34 (32.1)	11 (31.4)	28 (23.3)	
Varicocele [No. (%)]	48 (46.2)	50 (47.2)	18 (51.4)	62 (51.7)	0.83
FSH (mUI/mL)					<0.001
Median (IQR)	3.7 (2.4–6.1)	3.8 (2.8–4.5) ^#^	6.9 (3.7–10.2) ^§^^,^^†^	3.7 (2.6–5.4)	
Range	0.8–13.1	1.3–31.0	0.5–31.1	1.1–17.1	
LH (mUI/mL)					0.01
Median (IQR)	3.5 (2.4–4.3)	3.1 (2.4–4.6) ^#^	4.2 (3.1–6.0) ^§^	3.8 (2.7–5.1)	
Range	0.8–8.7	1.2–8.4	1.6–9.3	0.6–11.4	
InhB (pg/mL)					0.01
Median (IQR)	136.5(100.5–184.6)	147.3 (106.7–199.1) ^#^	105.2 (82.8–141.7) ^§^^,^^†^	141.0 (104.3–214.2)	
Range	16.9–334.8	14.0–326.7	8.0–285.1	6.0–538.2	
tT (ng/mL)					0.81
Median (IQR)	4.6 (3.6–5.5)	4.7 (3.8–5.8)	4.7 (3.4–6.1)	4.8 (3.3–5.9)	
Range	2.2–8.1	1.4–9.7	2.1–8.7	0.9–10.4	
SHBG (nmol/L)					0.96
Median (IQR)	33.0 (24–42)	32.0 (23–41)	28.9 (24–42)	32.0 (23–41)	
Range	9.0–85.0	11.0–99.0	14.0–154.0	11.0–86.0	
NLR					<0.01
Median (IQR)	1.5 (1.2–1.8)	1.9 (1.4–3.1) ^§^^,^^#^^,^^†^	1.4 (0.9–2.2)	1.7 (1.2–2.1)	
Range	0.7–5.7	0.7–5.1	0.7–9.6	0.7–3.5	
PLR					0.28
Median (IQR)	109.4 (86.1–135.1)	113.6 (90.6–147.5)	107.7 (81.6–128.7)	114.8 (87.3–140.5)	
Range	54.1–220.8	58.7–270.0	65.7–340.8	50.0–212.2	
MER					0.21
Median (IQR)	3.0 (2.0–4.0)	3.4 (2.0–5.0)	3.0 (2.5–5.0)	3.0 (2.0–4.3)	
Range	0.7–7.0	0.6–12.0	0.7–20.0	0.9–9.1	
Time of abstinence (days)					0.71
Median (IQR)	3.0 (2.0–4.0)	3.0 (2.0–5.0)	4.0 (2.0–4.0)	3.0 (2.0–5.0)	
Range	0.2–7.0	0.3–7.0	0.2–7.0	0.3–7.0	
Semen volume (mL)					0.12
Median (IQR)	3.0 (2.0–4.0)	3.0 (2.0–4.0)	4.0 (3.0–4.0)	3.0 (2.0–4.0)	
Range	0.1–8.0	0.1–8.0	0.1–10.0	0.1–10.0	
Sperm concentration					<0.001
Median (IQR)	44.3 (25.9–79.0)	48.2 (30.0–82.0)	6.0 (3.0–10.0)	37.3 (22.0–63.0)	
Range	15.1–339.0	16.2–214.8	0.5–12.3	15.0–350.2	
Progressive motility					<0.001
Median (IQR)	45.0 (38.0–54.0)	45.0 (37–52)	41.0 (36.0–55.0)	16.5 (10–27)	
Range	32.1–89.0	32.0–82.0	32.0–78.0	0.0–31.0	
Normal morphology					<0.001
Median (IQR)	12.5 (8–37)	1.0 (0.0–2.0)	15.0 (5–39)	8.5 (6.0–20.0)	
Range	4.0–90.0	0.0–3.0	4.0–82.0	5.0–100.0	
SDF index (%)					0.01
Median (IQR)	14.5 (5.3–24.3)	23.6 (12.9–42.5)^§^^,^^#^^,^^†^	12.7 (7.9–35.7)	13.6 (7.5–33.6)	
Range	0.5–95.3	3.8–93.2	0.4–96.4	0.5–90.0	
SDF index ≥ 30 [No. (%)]	17 (16.3)	49 (46.2)	8 (24.2)	31 (26.0)	0.02

Keys: SDF = Sperm DNA fragmentation; iTZS = Isolated teratozoospermia; iOZS = isolated oligozoospermia; iAZS = isolated asthenozoospermia; BMI = body mass index; CCI = Charlson Comorbidity Index; tT = total Testosterone; NLR = neutrophil-to-lymphocyte ratio; PLR = platelet-to-lymphocyte ratio; MER = monocyte-to-eosinophil ratio

* p value according to the Kruskal Wallis test and Fisher exact test, as indicated.

§ p < 0.001 vs. normal semen parameters group

# p < 0.001 vs. iOZS group

† p < 0.001 vs. iAZS group

**Fig 2:**
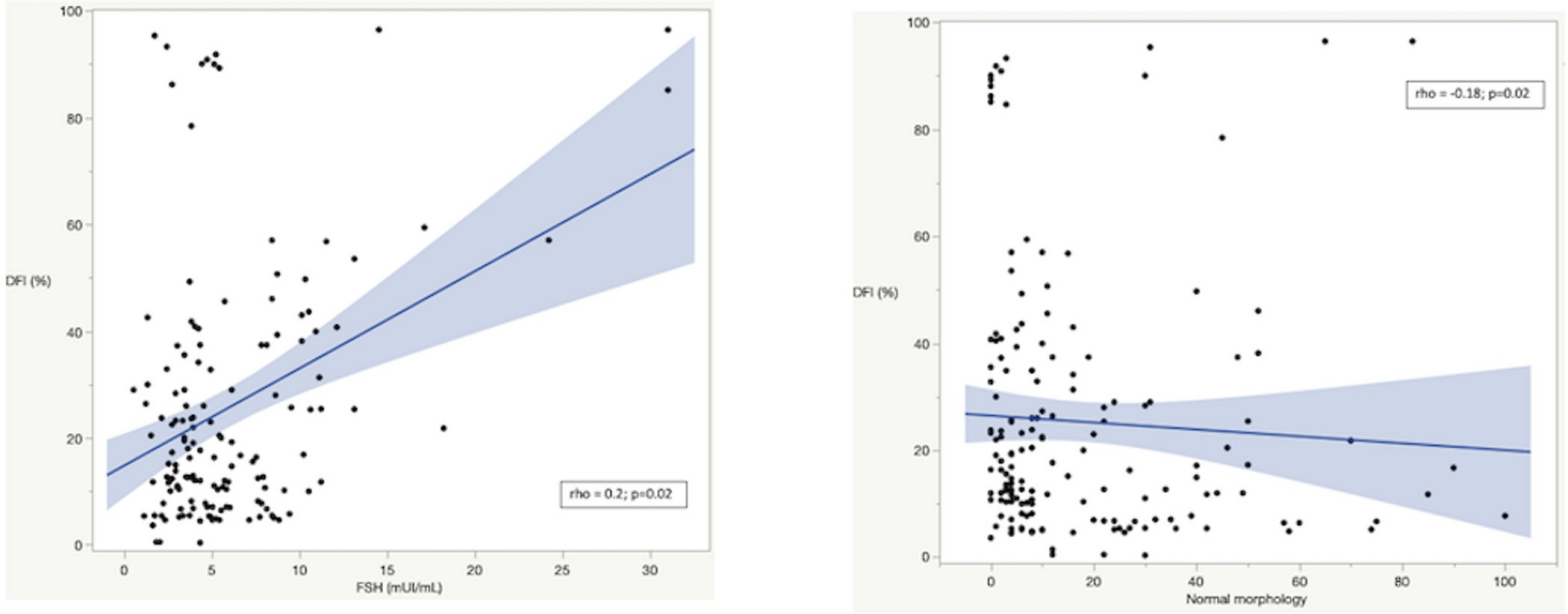
Correlation between sperm DNA fragmentation (SDF) and FSH values (A) and normal sperm morphology (B) in infertile men. Rho and p-value according to Spearman’s correlation.

**Fig 3:**
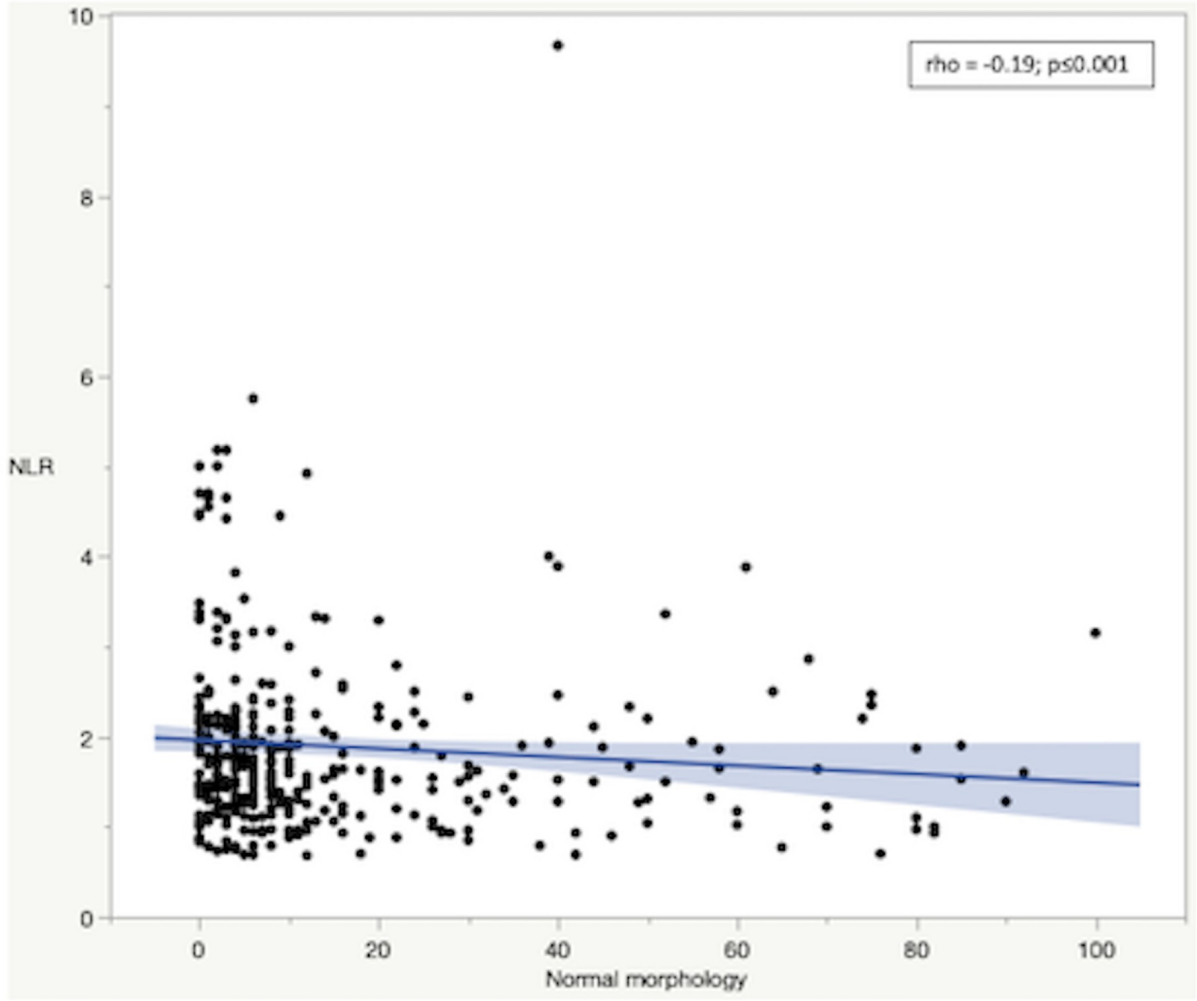
Correlation between Neutrophil-to-Lymphocyte Ratio (NLR) and normal sperm morphology in infertile men. Rho and p-value according to Spearman’s correlation.

In infertile men, Spearman’s correlation analysis revealed that SDF was positively associated with serum FSH values and negatively associated with sperm morphology (Fig 2A and 2B, respectively). Similarly, NLR was negatively associated with sperm morphology (Fig 3) and the higher NLR, the greater CCI (rho = 0.14; p<0.01). Conversely, NLR was not associated with sperm morphology in fertile men.

At multivariable analysis, CCI, smoking status, FSH and iTZS were associated with SDF (all p≤0.02), after accounting for age and sperm count (Table 3). Similarly, BMI, CCI, smoking status (all p<0.01) and iTZS (p<0.01) were associated with NLR, after accounting for age and sperm count (Table 3).

**Table 3:** Linear regression models predicting SDF and NLR (beta; *p* value [95%CI]) in infertile men.

	Sperm DNA fragmentation		Neutrophil-to-lymphocyte ratio	
	UVA model	MVA model	UVA model	MVA model
Age	0.42; 0.16	0.2; 0.76	1.12; 0.12	1.13; 0.34
	[-0.16–0.99]	[-0.44–0.77]	[-1.92–3.26]	[-1.92–3.11]
BMI	--	--	0.26; 0.02	0.37; 0.01
	--	--	[0.09–0.67]	[0.12–0.88]
CCI ≥ 1	3.72; 0.01	2.75; 0.02	0.85; <0.001	0.72; 0.01
	[1.27–6.43]	[1.12–4.85]	[0.46–1.29]	[0.34–1.08]
FSH	1.83; <0.001	1.73; <0.001	--	--
	[1.04–2.61]	[0.94–2.49]	--	--
Smoking status	4.32; <0.001	3.69; 0.01	0.53; 0.001	0.40; 0.01
Yes vs. No	[1.94–9.27]	[1.22–7.19]	[0.29–0.75]	[0.15–0.69]
Semen abnormalities				
No abnormalities	Ref	Ref	Ref	Ref
iTZS	2.87; <0.001	2.31; 0.01	0.52; <0.001	0.46; <0.01
	[1.1 –-7.13]	[0.88–6.39]	[0.26–0.79]	[0.21–0.75]
iOZS	-1.02; 0.31	-1.13; 0.75	0.24; 0.21	0.26; 0.18
	[-3.12–7.13]	[-2.11–5.26]	[-0.14–0.62]	[-0.11–0.62]
iAZS	1.21; 0.39	1.05; 0.82	0.1; 0.76	0.14; 0.93
	[-7.1–6.12]	[-2.13–4.35]	[-0.22–0.29]	[-0.25–0.28]
Sperm count	-0.12; <0.001	0.02; 0.55	-0.002; <0.001	-0.004; 0.98
	[-0.17–-0.07]	[-0.05–0.09]	[-0.003–-0.001]	[-0.001–0.08]

Keys: SDF = Sperm DNA fragmentation; NLR = neutrophil-to-lymphocyte ratio; UVA = Univariate model; MVA = Multivariate model, BMI = body mass index; CCI = Charlson Comorbidity Index; iTZS = Isolated teratozoospermia; iOZS = isolated oligozoospermia; iAZS = isolated asthenozoospermia;

Clinical and hormonal characteristics were similar between fertile and infertile men with iTZS (Table 4). Conversely, NLR was higher in infertile than fertile men (p<0.01). In the fertile cohort only, NLR, PLR and MER were comparable between iTZS men and those with normal semen parameters (Table 4).

**Table 4:** Descriptive statistics of participants with isolated teratozoospermia according to fertility status (No. = 143).

	Fertile	Infertile	p-value*
No. of individuals	37 (25.8%)	106 (74.1%)	
Age (years)			0.1
Median (IQR)	35.0 (33–38)	37.0 (34.0–41)	
Range	19–55	19–54	
BMI (kg/m^2^)			0.74
Median (IQR)	24.3 (22.6–26.7)	24.6 (23.0–26.5)	
Range	16.7–37.9	19.5–37.7	
CCI (score)			0.65
Median (IQR)	0.0 (0.0)	0.0 (0.0)	
Median (SD)	0.1 (0.1)	0.1 (0.3)	
Range	0–3	0–2	
CCI ≥ 1 [No. (%)]	3 (8.1)	9 (8.5)	0.54
Total testis volume (Prader estimation)			0.2
Median (IQR)	22.5 (20–25)	20.0 (15–25)	
Range	14–25	10–25	
Smoking status [No. (%)]			0.31
No smokers/Former smokers	28 (75.7)	72 (67.9)	
Active smokers	9 (24.3)	34 (32.1)	
Varicocele [No. (%)]	12 (32.4)	50 (47.2)	0.43
FSH (mUI/mL)			0.21
Median (IQR)	4.1 (3.1–5.3)	3.8 (2.8–4.5)	
Range	1.4–12.5	1.3–31.0	
LH (mUI/mL)			0.17
Median (IQR)	3.9 (2.5–5.0)	3.1 (2.4–4.6)	
Range	2.0–8.5	1.2–8.4	
tT (ng/mL)			0.54
Median (IQR)	5.1 (4.1–6.0)	4.7 (3.8–5.8)	
Range	2.4–9.3	1.4–9.7	
SHBG (nmol/L)			0.19
Median (IQR)	34.0 (31–46)	32.0 (23–41)	
Range	27.0–72.0	11.0–99.0	
NLR			<0.01
Median (IQR)	1.2 (0.8–3.3)	1.9 (1.4–3.1)	
Range	0.5–6.8	0.7–5.1	
PLR			0.76
Median (IQR)	117.8 (99.6–182.1)	113.6 (90.6–147.5)	
Range	12.8–230.1	58.7–270.0	
MER			0.29
Median (IQR)	3.3 (2.3–5.3)	3.4 (2.0–5.0)	
Range	0.7–15.0	0.6–12.0	
Time of abstinence (days)			0.56
Median (IQR)	3.0 (2.0–4.0)	3.0 (2.0–5.0)	
Range	0.2–7.0	0.3–7.0	
Semen volume (ml)			0.12
Median (IQR)	3.0 (2.0–4.0)	3.0 (2.0–4.0)	
Range	0.1–8.0	0.1–8.0	
Sperm concentration			<0.01
Median (IQR)	56.0 (32.6–84.0)	48.2 (30.0–82.0)	
Range	15.1–131.0	16.2–214.8	
Progressive motility			0.54
Median (IQR)	45.0 (39.0–55.0)	45.0 (37–52)	
Range	32.1–74.0	32.0–82.0	
Normal morphology			<0.01
Median (IQR)	2.0 (1.0–2.0)	1.0 (0.0–2.0)	
Range	1.0–3.0	0.0–3.0	

Keys: BMI = body mass index; CCI = Charlson Comorbidity Index; tT = total Testosterone;

NLR = neutrophil-to-lymphocyte ratio; PLR = platelet-to-lymphocyte ratio; MER = monocyte-to-eosinophil ratio.

* p value according to the Mann Whitney test and Fisher exact test, as indicated.

## Discussion

In a large cohort of white-European men searching medical help for primary couple’s infertility, we observed a 11.9% prevalence of iTZS. Likewise, we observed iAZS and iOZS in 13.9% and 4.1% of patients, respectively. Conversely, the prevalence of iTZS, iAZS and iOZS was 35.9%, 1% and 0, respectively, in the cohort of age-comparable fertile men. Therefore, counter-intuitively, our data suggests that iTZS was even more prevalent in fertile than infertile men. As far as we know, the current study is the first to investigate the rate of isolated sperm abnormalities in a cohort of fertile men.

Moreover, according to our baseline hypothesis, we found that infertile men with iTZS presented with greater parameters suggestive of inflammation and possible indirect signs of OS—as defined according to the NLR ratio and SDF—as compared with patients with either normal sperm parameters, iAZS, or even iOZS. Third, infertile men with iTZS presented with higher NLR values than fertile controls.

These findings confirm what previously reported by Belloc et al. [24], showing that approximately 25% of infertile men had an isolated sperm abnormality, with iAZS being the most prevalent sperm abnormality. Moreover, the high prevalence of iTZS in fertile men reported by our study is consistent with other reports showing that iTZS per se was not associated with decreased fertility [10, 25]. In this context, it is relevant to note that criticisms have been moved to the sperm morphological assessment criteria and to the impact of teratozoospermia toward reproductive outcomes [9]. As for circulating hormones, we observed that both FSH and LH were higher, while inhibin B was lower in infertile men presenting with iOZS compared to those in all other groups. These data further corroborate previous findings supporting a link between oligozoospermia and endocrine dysfunctions [26].

The correlations between sperm morphology, sperm DNA integrity and oxidative balance have been recently investigated [13, 27, 28]. In this context, it has been observed that several conditions including inflammatory and infectious diseases may increase systemic and seminal OS, thus leading to impaired sperm morphology [29–31]. Moreover, OS has been found to be closely related to MFI through sperm DNA and membrane damage [32, 33]. Overall, previous observations outlined that any inflammatory condition may increase neutrophil counts or OS related markers [34]. Recently, NLR, MER and PLR were found to be feasible and low-cost inflammatory biomarkers, increasingly used in the everyday clinical practice as prognostic factors in several general and uro/andrological diseased conditions [17, 18, 35–37]. Scarce data, however, is available regarding the link between systemic inflammation parameters, isolated semen abnormalities and sperm DNA damage. Sperm DNA integrity is a relatively recent marker of semen quality associated with both spontaneous and medically assisted pregnancy rates in clinical practice [1]. Previous data have suggested that infertile men have higher degree of sperm DNA damage than fertile controls [38] and that the extent of sperm DNA damage in infertile men increases along with the number of sperm abnormalities [38, 39]. For instance, in a study investigating factors affecting SDF among 1010 infertile Chinese men, Lu et al. found that SDF was negatively associated with sperm concentration, motility, total normal-progressively motile sperm count and the rate of normal morphology [40]. Our results corroborate these findings: the greater the number of sperm abnormalities, the higher the SDF in infertile men. Traditionally, SDF has been found to be negatively associated with sperm motility [41, 42], but recent evidence showing a negative association with sperm morphology are also emerging. In this direction, Sachdeva et al. analysed SDF and chromosomal aneuploidy in a cohort of 302 men from the United Arab Emirates [43]; after having subcategorized patients according to sperm parameters abnormalities, the authors found that men with teratozoospermia were among those with higher SDF values and greater degrees of sperm abnormalities compared to men with alterations in sperm motility or concentration. Brahem et al. reported an increased level of DNA fragmentation and a higher sperm chromosomal aneuploidy rate in infertile men with iTZS compared to fertile individuals [15]. Of relevance, DNA fragmentation was correlated to the incidence of sperm head and tail abnormalities. Similarly, Majzoub et al. analysed the association between sperm DNA fragmentation and sperm morphology in 1168 infertile men [44]. They found a direct negative correlation between sperm DNA fragmentation and normal sperm morphology, with a greater impact on sperm head defects. According to our baseline hypothesis, here we found that infertile men with iTZS had higher SDF values as compared with infertile men with either normal semen parameters or other isolated semen abnormalities. Therefore, in light of the strong association between iTZS, inflammatory status and elevated SDF values, we could speculate that, from a clinical standpoint, the finding of iTZS at semen analysis in infertile men should not be considered as a “mild alteration” and ignored but rather it should prompt a detailed characterization of the inflammatory status and the rate of DNA damage. In particular, men with iTZS could benefit of SDF testing and, when appropriate, of treatments able to reduce the level of sperm DNA damage [1].

Despite the aetiology of sperm DNA damage in infertile men is still not completely understood [45], improper packaging and ligation during sperm maturation [46], post-meiosis defective apoptosis [47] and OS are usually involved [48]. These mechanisms might either independently or co-dependently being responsible for sperm DNA damage in infertile men with iTZS. Recent studies have clearly shown that high seminal levels of ROS with decreased antioxidant enzymes were strongly correlated with increased SDF and sperm chromatin decondensation in men with iTZS [49]. Moreover, an association between TZS and sperm apoptosis has been also postulated [50].

Agarwal et al. suggested that spermatozoa with pathological morphology and activated leukocytes were the main source of ROS over-production in semen and that OS might further lead to structural cell defects [14]. Similarly, Ammar et al. found that ROS generated from morphologically abnormal spermatozoa along with impaired seminal antioxidant defence may eventually induce many changes in sperm cells, thus including membrane and DNA damage and cell death [13].

As a whole, sperm DNA defects as well as apoptosis and seminal OS can be interlinked in the context of TZS and may constitute a unified pathogenic molecular mechanism.

It is known that systemic inflammation affects spermatogenesis in infertile men, leading to defects in sperm morphology [32]. Besides, inflammatory diseases increase neutrophils in the circulation and consequently NLR values [51, 52]. Here we observed that NLR increased along with the number of sperm alterations in infertile men and that NLR values were higher in infertile patients with iTZS compared to those either with iOZS or iAZS or in men with normal semen parameters. Moreover, notwithstanding a counterintuitive greater prevalence of iTZS was found in fertile than in infertile men, sperm morphology per se correlates with NLR in infertile men only but not in the fertile counterpart. These findings help speculating that a higher oxidative distress is present in infertile men with iTZS than in both infertile men with other isolated semen abnormalities and in fertile individuals. On the one hand, the higher inflammatory status of iTZS men is mirrored by the higher NLR; on the other, the high neutrophil level on blood might also contribute to sperm abnormalities. It has been reported that oxidative mechanisms that eventually result in the production of reactive oxygen species, the release of several peptides or proteins and the formation of neutrophil extracellular traps have detrimental effects on seminal parameters [53]. Accordingly, high NLR could be considered as the consequence of oxidative stress which is responsible for sperm alterations and increased NLR itself might contribute to semen alterations. Therefore, current findings are novel compared to previous studies where an association between NLR and semen parameters was not observed [54, 55]. Important limitations of those studies were the small samples size and the lack of information about the fertility status of the normozoospermic control groups.

This significance of this study relies on: i) the large cohort of infertile men evaluated by a single expert uro/andrologist with a comprehensive diagnostic work-up; and, ii) the presence of a control group of fertile men with either normal and abnormal semen analyses [56], including iTZS, thus recapitulating the everyday clinical practice. Of utmost importance, this study is novel since it is the first to comprehensively compare infertile men according to isolated sperm abnormalities. In this context we showed that iOZS men had worse clinical and hormonal parameters but iTZS showed worse inflammatory biomarkers among groups.

However, our study has some limitations. First, it was a single academic center study, thus increasing the risk of selection biases. In particular, the numerical difference among fertile and infertile group might have influenced our results; thereof, larger studies across different centers and cohorts are needed to externally validate our findings. Second, the method employed to assess sperm morphology is very popular in infertility centers because it is a simple procedure that uses pre-colored slides and does not require the use of chemical reagents [57, 58], yet not reported by the WHO manual [5]. However, it has to be underlined that the procedure implies an External Quality Control (EQC) program coordinated by the Tuscany region (AOUC Careggi, Florence, Italy). Of note, the evaluation of the percentage of morphologically normal and abnormal spermatozoa was performed according to the WHO guidelines [5]. Third, our analysis lacked data regarding trends over time of inflammatory parameters, such as NLR. Forth, we lacked data regarding direct markers of semen OS in both cohorts, which may be of importance in the assessment of a common pathologic link fostering isolated semen abnormalities, inflammatory status and sperm DNA damage. However, direct testing of sperm markers of OS is not routine practice and it is not recommended by most of the current guidelines [1]. Lastly, we did not test sperm DNA damage in the cohort of fertile men.

## Conclusions

In this study, clinical, hormonal and seminal characteristics from infertile and fertile men with isolated sperm abnormalities were investigated. One out of ten men seeking medical help for primary couple’s infertility associated with a pure male factor presented with semen parameters suggestive for iTZS. While iTZS emerged to be even more prevalent among same-ethnicity, age-comparable fertile men, infertile men with iTZS had higher NLR than the fertile counterpart with isolated abnormal sperm morphology. Moreover, iTZS infertile men also depicted increased levels of sperm DNA fragmentation and of NLR than infertile individuals with both iAZS, iOZS, or normal semen parameters. Although weak, a significant association between sperm morphology, markers of systemic inflammation and sperm DNA damage in primary infertile men was also observed. Hence, the association between iTZS, inflammation and SDF impairment should hint the investigation of oxidative stress markers and sperm DNA quality in the everyday clinical practice.

## Supporting information

S1 Dataset (XLSX)

## References

[1] Salonia A, Bettocchi C, Carvalho J, Corona G, Jones TH, Kadioglu A, et al. EAU Guidelines on Sexual and Reproductive Health. 2020.

[2] Levine H, Jørgensen N, Martino-Andrade A, Mendiola J, Weksler-Derri D, Mindlis I, et al. Temporal trends in sperm count: A systematic review and meta-regression analysis. Hum Reprod Update. 2017 Nov 1;23(6):646–659. doi: 10.1093/humupd/dmx022 28981654 PMC6455044

[3] Geoffroy-Siraudin C, Dieudonné Loundou A, Romain F, Achard V, Courbière B, Perrard MH, et al. Decline of semen quality among 10 932 males consulting for couple infertility over a 20-year period in Marseille, France. Asian J Androl. 2012 ul;14(4):584–90. doi: 10.1038/aja.2011.173 22522503 PMC3724202

[4] Sengupta P, Borges E, Dutta S, Krajewska-Kulak E. Decline in sperm count in European men during the past 50 years. Hum Exp Toxicol. 2018 Mar;37(3):247–255. doi: 10.1177/0960327117703690 28413887

[5] Cooper TG, Noonan E, Eckardstein S von, Auger J, Baker HWG, Behre HM, et al. World Health Organization reference values for human semen characteristics. 2010 May-Jun;16(3):231–45. doi: 10.1093/humupd/dmp048 19934213

[6] Zhang T, Wu J, Liao C, Ni Z, Zheng J, Yu F. System analysis of teratozoospermia mRNA profile based on integrated bioinformatics tools. Mol Med Rep. 2018 Aug;18(2):1297–1304. doi: 10.3892/mmr.2018.9112 29901159 PMC6072217

[7] De Braekeleer M, Nguyen MH, Morel F, Perrin A. Genetic aspects of monomorphic teratozoospermia: a review. J Assist Reprod Genet. 2015 Apr;32(4):615–23. doi: 10.1007/s10815-015-0433-2 25711835 PMC4380889

[8] Menkveld R, Holleboom CAG, Rhemrev JPT. Measurement and significance of sperm morphology. Asian Journal of Andrology. 2011 Jan;13(1):59–68. doi: 10.1038/aja.2010.67 21076438 PMC3739393

[9] Danis RB, Samplaski MK. Sperm Morphology: History, Challenges, and Impact on Natural and Assisted Fertility. Current Urology Reports. 2019 Jun 15;20(8):43. doi: 10.1007/s11934-019-0911-7 31203470

[10] Hotaling JM, Smith JF, Rosen M, Muller CH, Walsh TJ. The relationship between isolated teratozoospermia and clinical pregnancy after in vitro fertilization with or without intracytoplasmic sperm injection: A systematic review and meta-analysis. Fertil Steril. 2011 Mar 1;95(3):1141–5. doi: 10.1016/j.fertnstert.2010.09.029 21030014

[11] Guzick DS, Overstreet JW, Factor-Litvak P, Brazil CK, Nakajima ST, Coutifaris C, et al. Sperm morphology, motility, and concentration in fertile and infertile men. N Engl J Med. 2001 Nov 8;345(19):1388–93. doi: 10.1056/NEJMoa003005 11794171

[12] Patel P, Carrasquillo R, Madhusoodanan V, Dadoun S, Patel A, Smith N, et al. Impact of Abnormal Sperm Morphology on Live Birth Rates Following Intrauterine Insemination. J Urol. 2019 Oct;202(4):801–805. doi: 10.1097/JU.0000000000000288 31009287

[13] Ammar O, Mehdi M, Muratori M. Teratozoospermia: Its association with sperm DNA defects, apoptotic alterations, and oxidative stress. Andrology. 2020 Sep;8(5):1095–1106. doi: 10.1111/andr.12778 32096605

[14] Agarwal A, Tvrda E, Sharma R. Relationship amongst teratozoospermia, seminal oxidative stress and male infertility. Reprod Biol Endocrinol. 2014 2014 May 27;12:45. doi: 10.1186/1477-7827-12-45 24884815 PMC4049374

[15] Brahem S, Mehdi M, Elghezal H, Saad A. Detection of DNA fragmentation and meiotic segregation in human with isolated teratozoospermia. J Assist Reprod Genet. 2011 Jan;28(1):41–8. doi: 10.1007/s10815-010-9482-8 20872065 PMC3045490

[16] Braham A, Ghedir H, Zidi I, Sallem A, Hajlaoui A, Ajina M, et al. Nuclear sperm quality in total polymorphic teratozoospermia and its impact on intracytoplasmic sperm injection outcome. Andrologia. 2019 Jun;51(5):e13252. doi: 10.1111/and.13252 30821000

[17] Cito G, Coccia ME, Picone R, Cocci A, Russo GI, Cai T, et al. Male inflammatory parameters are not useful to predict the outcomes of intracytoplasmic sperm injection: Results from a cross-sectional study. World J Mens Health. 2019 Sep;37(3):347–354. doi: 10.5534/wjmh.180110 30799563 PMC6704309

[18] Ventimiglia E, Cazzaniga W, Pederzoli F, Frego N, Chierigo F, Capogrosso P, et al. The role of neutrophil-to-lymphocyte ratio in men with erectile dysfunction—preliminary findings of a real-life cross-sectional study. Andrology. 2018 Jul;6(4):559–563. doi: 10.1111/andr.12489 29611369

[19] Rowe PJ, Comhaire FH, Hargreave TB, Mellows HJ. WHO Manual for the Standardized Investigation and Diagnosis of the Infertile Couple. 2000, Cambridge University.

[20] Charlson ME, Pompei P, Ales KL, MacKenzie CR. A new method of classifying prognostic comorbidity in longitudinal studies: development and validation. J Chronic Dis. 1987;40(5):373–83. doi: 10.1016/0021-9681(87)90171-8 3558716

[21] Ventimiglia E, Capogrosso P, Boeri L, Pederzoli F, Cazzaniga W, Scano R, et al. When to Perform Karyotype Analysis in Infertile Men? Validation of the European Association of Urology Guidelines with the Proposal of a New Predictive Model. Eur Urol. 2016 Dec;70(6):920–923. doi: 10.1016/j.eururo.2016.06.015 27343001

[22] Evenson DP, Larson KL, Jost LK. Sperm chromatin structure assay: Its clinical use for detecting sperm DNA fragmentation in male infertility and comparisons with other techniques. Journal of Andrology. 2002 Jan-Feb;23(1):25–43. doi: 10.1002/j.1939-4640.2002.tb02599.x 11780920

[23] Alharbi M, Hamouche F, Phillips S, Kadoch J, Zini A. Use of testicular sperm in couples with SCSA-defined high sperm DNA fragmentation and failed intracytoplasmic sperm injection using ejaculated sperm. Asian J Androl. 2020 Jul-Aug;22(4):348–353. doi: 10.4103/aja.aja_99_19 31571640 PMC7406103

[24] Belloc S, Benkhalifa M, Cohen-Bacrie M, Dalleac A, Chahine H, Amar E, et al. Which isolated sperm abnormality is most related to sperm DNA damage in men presenting for infertility evaluation. J Assist Reprod Genet. 2014 May;31(5):527–32. doi: 10.1007/s10815-014-0194-3 24566945 PMC4016368

[25] Kovac J, Smith R, Cajipe M, Lamb D, Lipshultz L. Men with a complete absence of normal sperm morphology exhibit high rates of success without assisted reproduction. Asian J Androl. 2017 Jan-Feb;19(1):39–42. doi: 10.4103/1008-682X.189211 27751992 PMC5227671

[26] Choy JT, Amory AJ. Nonsurgical Management of Oligozoospermia. J Clin Endocrinol Metab. 2020 Dec 1;105(12):e4194–207. doi: 10.1210/clinem/dgaa390 32583849 PMC7566408

[27] Muratori M, Tamburrino L, Marchiani S, Cambi M, Olivito B, Azzari C, et al. Investigation on the origin of sperm DNA fragmentation: Role of apoptosis, immaturity and oxidative stress. Mol Med. 2015 Jan 30;21(1):109–22. doi: 10.2119/molmed.2014.00158 25786204 PMC4461587

[28] Cocuzza M, Sikka SC, Athayde KS, Agarwal A. Clinical relevance of oxidative stress and sperm chromation damage in male infertility: An evidence based analysis. International Braz J Urol. 2007 Sep-Oct;33(5):603–21. doi: 10.1590/s1677-55382007000500002 17980058

[29] Agarwal A, Rana M, Qiu E, AlBunni H, Bui AD, Henkel R. Role of oxidative stress, infection and inflammation in male infertility. Andrologia. 2018 Dec;50(11):e13126. doi: 10.1111/and.13126 30569652

[30] Azenabor A, Ekun AO, Akinloye O. Impact of inflammation on male reproductive tract. Journal of Reproduction and Infertility. 2015 Jul-Sep;16(3):123–9. 26913230 PMC4508350

[31] Boeri L, Capogrosso P, Ventimiglia E, Pederzoli F, Cazzaniga W, Chierigo F, et al. High-risk human papillomavirus in semen is associated with poor sperm progressive motility and a high sperm DNA fragmentation index in infertile men. Hum Reprod. 2019 Feb 1;34(2):209–217. doi: 10.1093/humrep/dey348 30517657

[32] Barati E, Nikzad H, Karimian M. Oxidative stress and male infertility: current knowledge of pathophysiology and role of antioxidant therapy in disease management. Cellular and Molecular Life Sciences. 2020 Jan;77(1):93–113. doi: 10.1007/s00018-019-03253-8 31377843 PMC11105059

[33] Bisht S, Faiq M, Tolahunase M, Dada R. Oxidative stress and male infertility. Nature Reviews Urology. 2017 Aug;14(8):470–485. doi: 10.1038/nrurol.2017.69 28508879

[34] Kotani K, Sakane N. White blood cells, neutrophils, and reactive oxygen metabolites among asymptomatic subjects. Int J Prev Med. 2012 Jun;3(6):428–31. 22783470 PMC3389441

[35] Forget P, Khalifa C, Defour JP, Latinne D, Van Pel MC, De Kock M. What is the normal value of the neutrophil-to-lymphocyte ratio? BMC Res Notes. 2017 Jan 3;10(1):12. doi: 10.1186/s13104-016-2335-5 28057051 PMC5217256

[36] Zahorec R. Ratio of neutrophil to lymphocyte counts—rapid and simple parameter of systemic inflammation and stress in critically ill. Bratisl Lek Listy. 2001 2001;102(1):5–14. 11723675

[37] Imtiaz F, Shafique K, Mirza S, Ayoob Z, Vart P, Rao S. Neutrophil lymphocyte ratio as a measure of systemic inflammation in prevalent chronic diseases in Asian population. Int Arch Med. 2012 Jan 26;5(1):2. doi: 10.1186/1755-7682-5-2 22281066 PMC3277482

[38] Zini A, Fischer MA, Sharir S, Shayegan B, Phang D, Jarvi K. Prevalence of abnormal sperm DNA denaturation in fertile and infertile men. Urology. 2002 Dec;60(6):1069–72. doi: 10.1016/s0090-4295(02)01975-1 12475672

[39] Moskovtsev SI, Willis J, White J, Mullen JBM. Sperm DNA Damage: Correlation to Severity of Semen Abnormalities. Urology. 2009 Oct;74(4):789–93. doi: 10.1016/j.urology.2009.05.043 19643462

[40] Lu JC, Jing J, Chen L, Ge YF, Feng RX, Liang YJ, et al. Analysis of human sperm DNA fragmentation index (DFI) related factors: A report of 1010 subfertile men in China. Reprod Biol Endocrinol. 2018 Mar 14;16(1):23. doi: 10.1186/s12958-018-0345-y 29540184 PMC5852972

[41] Le MT, Nguyen TAT, Nguyen HTT, Nguyen TTT, Nguyen VT, Le DD, et al. Does sperm DNA fragmentation correlate with semen parameters? Reprod Med Biol. 2019 Sep 3;18(4):390–396. doi: 10.1002/rmb2.12297 31607800 PMC6780033

[42] Ganzer LM, Larcher JMS, Avramovich VI, Tissera AD, Estofan GM. Relationship between semen parameters and sperm DNA fragmentation. Fertil Steril. 2017 10.1016/j.fertnstert.2017.07.415.

[43] Sachdeva K, Upadhyay D, Neri JG, Varghese MM, Singh K, Albuz FK, et al. Semen Quality is Associated with Sperm Aneuploidy and DNA Fragmentation in the United Arab Emirates Population. Genet Test Mol Biomarkers. 2020 Apr;24(4):195–203. doi: 10.1089/gtmb.2019.0180 32208936

[44] Majzoub A, Arafa M, Mahdi M, Agarwal A, Al Said S, Al-Emadi I, et al. Oxidation–reduction potential and sperm DNA fragmentation, and their associations with sperm morphological anomalies amongst fertile and infertile men. Arab J Urol. 2018 Feb 1;16(1):87–95. doi: 10.1016/j.aju.2017.11.014 29713539 PMC5922185

[45] Schulte RT, Ohl DA, Sigman M, Smith GD. Sperm DNA damage in male infertility: Etiologies, assays, and outcomes. Journal of Assisted Reproduction and Genetics. 2010 Jan;27(1):3–12. doi: 10.1007/s10815-009-9359-x 20012685 PMC2826626

[46] Zini A, Libman J. Sperm DNA damage: Clinical significance in the era of assisted reproduction. CMAJ. 2006 Aug 29;175(5):495–500. doi: 10.1503/cmaj.060218 16940270 PMC1550758

[47] Sakkas D, Mariethoz E, Manicardi G, Bizzaro D, Bianchi PG, Bianchi U. Origin of DNAdamage in ejaculated human spermatozoa. Reviews of Reproduction. 1999 Jan;4(1):31–7. doi: 10.1530/ror.0.0040031 10051100

[48] Aitken RJ, Bronson R, Smith TB, De Iuliis GN. The source and significance of dna damage in human spermatozoa; a commentary on diagnostic strategies and straw man fallacies. Mol Hum Reprod. 2013 Aug;19(8):475–85. doi: 10.1093/molehr/gat025 23548339

[49] Oumaima A, Tesnim A, Zohra H, Amira S, Ines Z, Sana C, et al. Investigation on the origin of sperm morphological defects: oxidative attacks, chromatin immaturity, and DNA fragmentation. Environ Sci Pollut Res. 2018 May;25(14):13775–13786. doi: 10.1007/s11356-018-1417-4 29508198

[50] Aziz N, Said T, Paasch U, Agarwal A. The relationship between human sperm apoptosis, morphology and the sperm deformity index. Hum Reprod. 2007 May;22(5):1413–9. doi: 10.1093/humrep/dem016 17303629

[51] Rosales C. Neutrophil: A cell with many roles in inflammation or several cell types? Frontiers in Physiology. 2018 Feb 20;9:113. doi: 10.3389/fphys.2018.00113 29515456 PMC5826082

[52] Selders GS, Fetz AE, Radic MZ, Bowlin GL. An overview of the role of neutrophils in innate immunity, inflammation and host-biomaterial integration. Regen Biomater. 2017 Feb;4(1):55–68. doi: 10.1093/rb/rbw041 28149530 PMC5274707

[53] Hermosilla C, Caro TM, Silva LM, Ruiz A TA. The intriguing host innate immune response: novel anti-parasitic defence by neutrophil extracellular traps. Parasitology. 2014 Sep;141(11):1489–98. doi: 10.1017/S0031182014000316 24721985

[54] Aykan S, Canat L, Gönültaş S, Atalay HA, Altunrende F. Are There Relationships between Seminal Parameters and the Neutrophil-to-Lymphocyte Ratio or the Platelet-to-Lymphocyte Ratio? World J Mens Health. 2017 Apr;35(1):51–56. doi: 10.5534/wjmh.2017.35.1.51 28459146 PMC5419115

[55] Öztekin Ü, Caniklioğlu M, Sarı S, Selmi V, Gürel A, Caniklioğlu A, et al. Are There Any Relationships Between Abnormal Seminal Parameters and Neutrophil-Lymphocyte Ratio, Platelet-Lymphocyte Ratio, and Red Blood Cell Distribution-Platelet Ratio? Cureus. 2019 Jul 25;11(7):e5242. doi: 10.7759/cureus.5242 31565640 PMC6759139

[56] Boeri L, Belladelli F, Capogrosso P, Cazzaniga W, Candela L et al. Normal sperm parameters per se do not reliably account for fertility: A case–control study in the real-lifesetting. Andrologia. 2020 Feb;53(1):e13861. doi: 10.1111/and.13861 33125742

[57] Schirren C, Eckhardt U, Jachczik R, Carstensen CA. Morphological differentiation of human spermatozoa with Testsimplets slides. Andrologia. 1977 Apr-Jun;9(2):191–2. doi: 10.1111/j.1439-0272.1977.tb01283.x 70178

[58] Ombelet W, Pollet H, Bosmans E, Vereecken A. Results of a questionnaire on sperm morphology assessment. Hum Reprod. 1997 May;12(5):1015–20. doi: 10.1093/humrep/12.5.1015 . 9194657

